# ChatTogoVar: a TogoVar-based retrieval-augmented generation system for precise genomic variant interpretation

**DOI:** 10.1038/s41439-026-00344-4

**Published:** 2026-04-09

**Authors:** Nobutaka Mitsuhashi, Toyofumi Fujiwara, Atsuko Yamaguchi

**Affiliations:** 1 Database Division for Life Science, BioData Science Initiative, National Institute of Genetics, Research Organization of Information and Systems, 178-4-4 Wakashiba, Kashiwa, Chiba 277-0871 Japan; 2 Graduate School of Integrative Science and Engineering, Tokyo City University, 1-28-1 Tamazutsumi, Setagaya-ku, Tokyo 158-8557 Japan

**Keywords:** Genetic databases, Data mining

## Abstract

Large language models (LLMs) have recently been adopted to assist in the interpretation of human genomic variants. However, general-purpose LLMs can produce incorrect outputs (commonly termed ‘hallucinations’), particularly on specialized queries, raising concerns about their reliability for variant interpretation. Here, to mitigate this risk, we developed ChatTogoVar, a retrieval-augmented generation system that queries TogoVar, a variant database that integrates information, such as allele frequency and clinical significance, and incorporates the retrieved results into prompts. We constructed a benchmark of 150 questions sampled from a predefined pool of 1500 template–variant combinations (50 templates × 30 variants). For large-scale assessment, we used the full 1500-question pool for automated LLM-based scoring. ChatTogoVar achieved the highest score for 135/150 questions, outperforming both a general-purpose LLM and an existing specialized system. Furthermore, automatic evaluation of all 1500 questions by an LLM confirmed the same trend. These results suggest that integrating a reliable variant database with an LLM can improve the accuracy of variant interpretation and that ChatTogoVar may serve as a practical tool to support genomic medicine and personalized healthcare.

## ChatTogoVar facilitates genomic variant interpretation without compromising accuracy

As genomic medicine evolves, interpreting human variants becomes crucial, with variant databases such as TogoVar, gnomAD and ClinVar playing a key role. These databases help to determine variant frequency, disease association and interpretation reliability. However, accessing this information can be challenging for non-experts. The study introduces ChatTogoVar, a question-answering system that uses retrieval-augmented generation to reduce inaccuracies by integrating data from TogoVar, a comprehensive Japanese genomic variant database. ChatTogoVar was evaluated against general-purpose language models and VarChat, showing superior performance in accuracy and evidence support. It effectively integrates variant information, such as allele frequencies and clinical significance, from trusted databases. This approach minimizes errors and enhances genomic variant interpretation, crucial for personalized medicine. Future directions include expanding database integration to cover pharmacogenomics and complex queries, enhancing the system’s usability and reliability.

This summary was initially drafted using artificial intelligence, then revised and fact-checked by the author.

## Introduction

As genomic medicine advances, interpretation of human variants has become increasingly important^1^, and variant databases are central to this task^2^. These databases are widely used as information resources not only by researchers and clinicians but also by genetic counselors, laboratory technicians and other healthcare professionals involved in genomic medicine. For example, after identifying a variant that may cause disease, the next information needed is how often the variant is observed in the general population (allele frequency), whether its association with disease has been reported (clinical significance) and the reliability of its interpretation (evidence from literature or expert classification). To comprehensively understand these aspects, variant databases, such as TogoVar^3^, gnomAD^4^ and ClinVar^5^, are indispensable. TogoVar, launched in 2018, integrates variant information with an emphasis on allele frequencies in the Japanese population and serves as a fundamental resource for analyzing genomic variants in Japan.

An estimate projects that >60 million patients worldwide will undergo clinical genome analysis by 2025^6^. Hence, opportunities for healthcare professionals to access variant information will continue to increase. Traditionally, users of variant databases, including TogoVar, had to search for the necessary information manually by referring to help pages, a process that can be challenging for non-experts. However, the recent use of large language models (LLMs) in chat-based systems has begun to address this challenge^7^. LLMs can provide conversational and contextualized summaries in response to natural language queries. For example, an LLM asked ‘Is this variant associated with diabetes?’ can produce a concise answer with background and supporting citations.

General-purpose LLMs, such as GPT-4 Omni (GPT-4o)^8^, can provide answers to natural language questions. However, a fundamental limitation of such models is ‘hallucination’ (the generation of plausible but incorrect information)^9^. For instance, a model may incorrectly respond that no association exists even when one has been reported. Given the clinical significance of a patient’s genomic variant in case reports and the need for high accuracy, hallucinations pose a serious problem in this application^10,11^.

One attempt to mitigate hallucinations in variant interpretation is VarChat^12^. VarChat provides LLM-generated summaries of publications indexed in PubMed and freely available through Google Scholar. It also provides links to source articles and ClinVar for variants specified by a reference single-nucleotide polymorphism (SNP) number (rs number), a Human Genome Variation Society (HGVS) notation^13^, a gene symbol or a genomic position. Its design makes it easier for non-expert users to access the information needed for variant interpretation. In addition, it presents pregenerated answers to frequently asked questions about each variant. However, its scope is limited to variants reported in the literature, resulting in low coverage and limited flexibility to handle arbitrary free-text queries.

To address the limitations of LLMs in genomic variant interpretation, we developed ChatTogoVar, a question-answering system based on the retrieval-augmented generation (RAG) framework, which reduces hallucinations by retrieving factual information from trusted databases and incorporating it into LLM prompts. In ChatTogoVar, we adopted TogoVar—a comprehensive Japanese genomic variant database integrating over 800 million variants from large-scale datasets and literature—as the external knowledge source, providing broader coverage than text-mining resources such as PubTator3^14^, which identify about 2 million variants from publications. To evaluate system performance, we compared ChatTogoVar with a general-purpose LLM (GPT-4o) and a variant-specific system (VarChat). The benchmark comprised 150 manually evaluated questions and 1,500 automatically scored ones, both assessed on criteria such as accuracy and evidence support. ChatTogoVar consistently achieved higher scores across evaluations, demonstrating that integrating reliable databases through the RAG framework effectively reduces hallucinations and enhances reliability in genomic variant interpretation, contributing to the advancement of genomic and personalized medicine.

## Materials and methods

The first subsection explains the development of the TogoVar application programming interface (API), the second subsection describes the architecture of ChatTogoVar for generating answers to user questions, and the third subsection outlines the evaluation design used to assess its performance.

### Development of TogoVar API

**Fig. 1:**
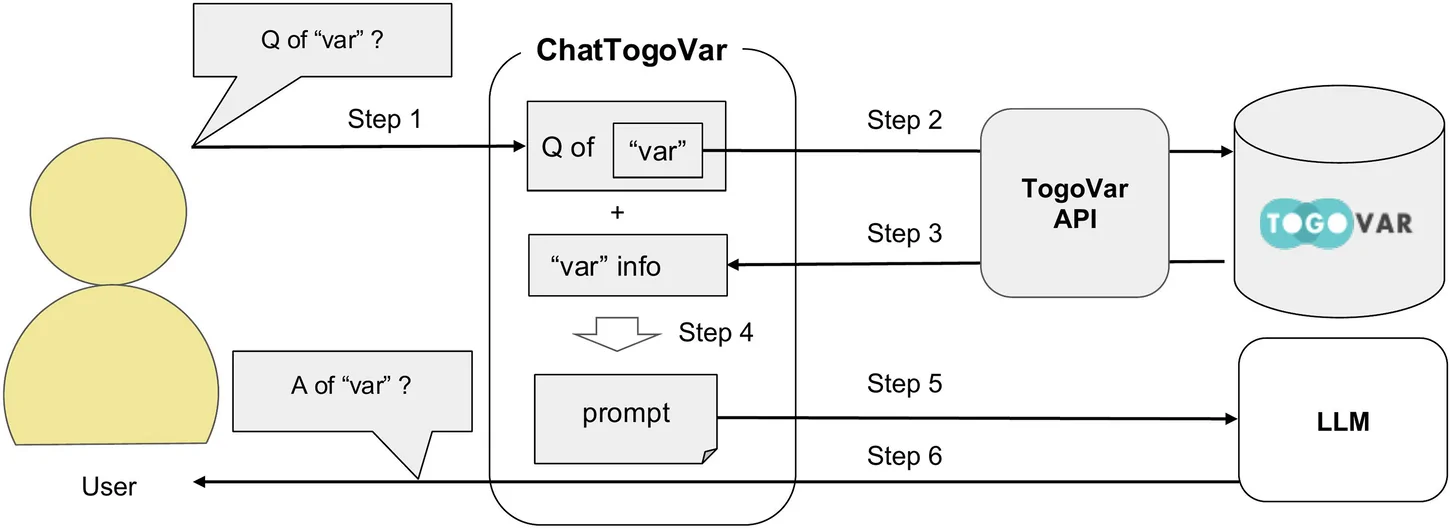
Architecture of ChatTogoVar. ChatTogoVar integrates user queries with information retrieved from the TogoVar API and the knowledge of a LLM. Through a multistep RAG process, the system generates comprehensive answers that combine database-derived evidence with LLM-based reasoning to reduce hallucinations and improve accuracy in variant interpretation.

We developed the TogoVar API, a key RAG module designed to mitigate hallucinations; this enables ChatTogoVar to query the external database TogoVar and retrieve reliable information (Fig. 1). The API returns data for a specified rs number in JSON format. An example query and response are shown in Supplementary Fig. 1, and the variant attributes included in the response are summarized in Table 1.

**Table 1:** Summarized information for rs34637584 obtained via the TogoVar API.

Field	Description	Example
ID	TogoVar variant identifier	tgv45580587
Existing variation	dbSNP identifier	rs34637584
Chromosome	Chromosome number	12
Position	Genomic position on GRCh38	40340400
Reference allele	Reference allele (REF)	G
Alternate allele	Alternate allele (ALT)	A
Gene symbol	Gene symbol associated with the variant	LRRK2
Clinical significance	Clinical interpretation and related condition(s)	Autosomal dominant Parkinson disease 8 (pathogenic, likely pathogenic)
Most severe consequence	Most impactful consequence term by Sequence Ontology	SO_0001583 (missense variant)
HGVS nomenclature	HGVS description at protein, cDNA and genomic levels	ENSP00000298910.7:p.Gly2019Ser, ENST00000298910.12:c.6055G>A, chr12:g.40340400G>A
Pathogenicity prediction	Deleterious prediction score by SIFT, PolyPhen and AlphaMissense	SIFT: 0 (deleterious), PolyPhen: 1 (probably damaging), AlphaMissense: 0.936 (likely pathogenic)
Allele frequency	Allele frequencies in various databases and populations	ToMMo: 0.000028, gnomAD Genomes: 0.00036, gnomAD Exomes: 0.00041, GEM-J WGA: 0.000065, NCBN: 0.00012
External link	Links to ClinVar, gnomAD, dbSNP and so on	https://www.ncbi.nlm.nih.gov/clinvar/variation/1940https://gnomad.broadinstitute.org/variant/12-40340400-G-A?dataset=gnomad_r4https://identifiers.org/dbsnp/rs34637584

### Architecture of ChatTogoVar

We designed the architecture of ChatTogoVar to generate answers using the RAG approach, as illustrated in Fig. 1. A variety of LLM platforms can be applied to the architecture, including cloud-based platforms, such as Azure OpenAI^15^ and Google Gemini^16^, as well as self-hosted platforms, such as Meta’s Llama^17^ and OpenAI’s GPT-OSS^18^ series. The processing of a user’s question proceeds as follows:

#### Step 1

The user inputs a question, such as ‘What is the clinical significance of rs34637584 in ClinVar?’.

#### Step 2

ChatTogoVar extracts an rs number or HGVS notation from the question and sends it as a query to the TogoVar API. If an HGVS notation is detected, it is passed to Ensembl Variant Recoder^19^ to obtain a GRCh38 genomic representation (chromosome, 1-based position, reference allele and alternate allele), which is then used for the TogoVar API query. Otherwise, the extracted rs number is sent directly. A sample query is shown in Supplementary Fig. 1a. If neither an rs number nor HGVS notation is found in the question, then the user is directed to step 5.

#### Step 3

The TogoVar API returns information for the queried variant (that is, the rs number, or the GRCh38-normalized representation converted from an HGVS notation) in JSON format. A sample response is shown in Supplementary Fig. 1b, and the variant attributes included in the response are summarized in Table 1.

#### Step 4

ChatTogoVar constructs a prompt to be sent to the LLM. The prompt follows the format shown in the ‘Prompt template for ChatTogoVar’ section below, with the placeholder {togovar_response} replaced by the JSON obtained in step 3.

#### Step 5

ChatTogoVar sends the user’s question with the constructed prompt to the LLM.

#### Step 6

ChatTogoVar returns the answer generated by the LLM to the user.

### Prompt template for ChatTogoVar

A well-designed prompt is essential for instructing an LLM to perform tasks appropriately. The prompt template customized for ChatTogoVar consists of three sections. In the first section, ‘Expert assistant role’, ChatTogoVar specifies that the LLM should generate an answer by combining both the information retrieved from the TogoVar API and the knowledge already encoded in the LLM. The second section, ‘Items to include in the answer’, specifies what should be included as supporting evidence, enabling users to verify the returned answer. If no relevant supporting data are found, the system explicitly states this to prevent hallucinations. In the final section, ‘Information retrieved from the TogoVar API’, the JSON retrieved from the API is appended to the end of the prompt. The complete template is shown below.

### Expert assistant role

You are an expert assistant specializing in human genome variant analysis. When answering, generate your response based on both the information retrieved from the TogoVar API and your own knowledge. However, if the retrieved results are insufficient or do not fully address the question, use your own knowledge and reasoning to provide a comprehensive answer. Clearly indicate when your response is based on external knowledge rather than TogoVar API data.

### Items to include in the answer

Provide information on the following items (1–6) if directly relevant to the question. If no information is available, explicitly state that there are no data. Include a source URL for each answer whenever applicable.

1. Variant identification: rs number, HGVS notation, gene name and transcript name with links where possible;
2. Disease associations: relationships with diseases based on curated databases (ClinVar) and predictive models (AlphaMissense, SIFT, PolyPhen);
3. Literature evidence: relevant publications where this variant has been mentioned;
4. Allele frequency comparison: allele frequencies in Japanese versus non-Japanese populations, with explanation of population-specific differences if possible;
5. GWAS findings: relevant phenotypes associated with this variant based on genome-wide association studies (GWAS);
6. TogoVar link: direct link to the variant page in TogoVar (https://togovar.org).

### Information Retrieved from the TogoVar API

{togovar_response}

### Evaluation design

We compared three systems (ChatTogoVar, GPT-4o and VarChat) under two evaluation settings (manual and LLM-based), where ‘system’ denotes the evaluated response-generation pipeline and ‘evaluation setting’ denotes the scoring framework applied to its outputs. To ensure both reliability and scalability, we combined manual expert assessment with large-scale LLM-based scoring. Starting from a manually assessed set of 150 questions, we scaled the evaluation 10-fold to 1500 questions using GPT-4o as an automated evaluator, greatly enhancing variant coverage and statistical power. The details are described below.

### Comparison with GPT-4o and VarChat

The difference between ChatTogoVar and GPT-4o is whether knowledge from the TogoVar API is utilized. Thus, apart from the instruction to query TogoVar and include the retrieved results in the prompt, both systems were provided with the same prompt text. The LLM used for ChatTogoVar and GPT-4o was a GPT-4o model (version 2025-02-01-preview) provided by Azure OpenAI and accessed via the Chat Completions API^20^. VarChat, by contrast, accepts only a variant identifier and always returns the same predefined response, independent of the input question. VarChat was selected as a representative example of a simple variant-based chat system.

### Evaluation criteria

We used GPTScore^21^, an LLM-based evaluation method that scores responses in terms of accuracy, completeness and logical consistency, as the basis for our evaluation framework. Because GPTScore was originally developed for dialogue response generation, text summarization, data-to-text generation and machine translation, we modified it for the context of database-driven question answering. Accordingly, we defined the following five evaluation criteria to assess the answers generated by ChatTogoVar:

- *Accuracy* (*GPTScore: accuracy, ACC*)
  How accurately does the response address the question?
- *Completeness* (*GPTScore: semantic coverage, COV*)
  Does the response itself provide all the necessary information required to fully answer the question, rather than just pointing to potential sources?
- *Logical consistency* (*GPTScore: consistency, CON*)
  Does the response maintain logical coherence, with no contradictions?
- *Clarity and conciseness* (*GPTScore: coherence, COH*)
  Is the response clear and concise, with no ambiguity?
- *Evidence support* (*GPTScore: factuality, FAC*)
  Does the response rely on credible sources or evidence?

### Manual evaluation and question generation

To evaluate the systems, we first constructed a set of questions covering major aspects of variant interpretation. Questions were designed to assess allele frequency, functional impact and pathogenicity prediction, corresponding to elements of the evidence framework defined in the American College of Medical Genetics and Genomics and the Association for Molecular Pathology guidelines^22^. To expand the coverage, we added questions on known clinical significance, variant–gene relationships, pharmacogenomics, evolutionary context, relationships with other variants, and databases or analysis tools, resulting in 50 question templates grouped into 8 categories (Table 2).

**Table 2:** Fifty questions for system evaluation.

No.	Category	Question statement
q1	Basic information	Tell me the basic information about {rs}.
q2	Basic information	What is the genomic location of {rs} in GRCh38 and GRCh37?
q3	Basic information	Which gene is associated with {rs}?
q4	Basic information	What is the reference allele and alternative allele of {rs}?
q5	Basic information	Is {rs} a single nucleotide variant (SNV), insertion, or deletion?
q6	Basic information	What is the minor allele frequency (MAF) of {rs}?
q7	Basic information	Where can I find genomic data for {rs}?
q8	Basic information	What is the Hardy-Weinberg equilibrium status of {rs} in various populations?
q9	Allele frequency and population distribution	Could you show me the allele frequency of {rs} in Japanese populations?
q10	Allele frequency and population distribution	How does the allele frequency of {rs} compare across different populations?
q11	Allele frequency and population distribution	What is the allele frequency of {rs} in East Asian populations?
q12	Allele frequency and population distribution	What is the allele frequency of {rs} in European populations?
q13	Allele frequency and population distribution	What is the allele frequency of {rs} in African populations?
q14	Allele frequency and population distribution	Has {rs} undergone recent positive selection in any population?
q15	Allele frequency and population distribution	Is there any evidence of natural selection acting on {rs}?
q16	Clinical significance	Is {rs} associated with any diseases or clinical conditions?
q17	Clinical significance	What is the clinical significance of {rs} in ClinVar?
q18	Clinical significance	How does the location of {rs} influence the clinical phenotype?
q19	Clinical significance	Is {rs} listed in any GWAS studies?
q20	Clinical significance	Does {rs} increase genetic risk for any diseases?
q21	Clinical significance	How does {rs} influence cancer risk?
q22	Clinical significance	What are the latest clinical findings about {rs}?
q23	Clinical significance	How does {rs} contribute to polygenic risk scores (PRS)?
q24	Pharmacogenomics	Does {rs} influence drug metabolism or response?
q25	Pharmacogenomics	What pharmacogenomic associations exist for {rs}?
q26	Pharmacogenomics	How does {rs} affect the metabolism of alcohol or specific drugs?
q27	Pharmacogenomics	Is {rs} included in any pharmacogenomics guidelines (e.g., PharmGKB)?
q28	Pharmacogenomics	Can {rs} be used as a biomarker for drug response?
q29	Functional impact	How does the {rs} allele affect the structure and function of genes?
q30	Functional impact	What molecular mechanisms are affected by {rs}?
q31	Functional impact	How does {rs} affect transcript expression?
q32	Functional impact	What experimental evidence exists for {rs}’s functional impact?
q33	Functional impact	How does {rs} affect splicing regulation?
q34	Functional impact	Can {rs} be a target for genome editing (CRISPR)?
q35	Functional impact	Is {rs} a dominant or recessive variant?
q36	Evolutionary context	What is the evolutionary background of {rs}?
q37	Evolutionary context	What is the ancestral allele of {rs}?
q38	Evolutionary context	How does {rs} interact with environmental factors?
q39	Evolutionary context	What are the effects of {rs} in cis-acting elements?
q40	Evolutionary context	What selection pressures have acted on {rs} over time?
q41	Comparison with related variants	Tell me about variants that have similar effects to the {rs} variant.
q42	Comparison with related variants	Are there novel variants with similar effects to {rs}?
q43	Comparison with related variants	What haplotypes include {rs}?
q44	Comparison with related variants	How is {rs} different from other known variants in the same gene?
q45	Comparison with related variants	What other missense variants exist in the same gene as {rs}?
q46	Comparison with related variants	Are there any compensatory mutations that counteract the effect of {rs}?
q47	Databases and bioinformatics analysis	What bioinformatics tools can analyze {rs}?
q48	Databases and bioinformatics analysis	What datasets contain information on {rs}?
q49	Databases and bioinformatics analysis	Is {rs} included in any population genomics databases (e.g., gnomAD, 1000 Genomes)?
q50	Databases and bioinformatics analysis	What machine learning models have been used to predict the impact of {rs}?

The questions are grouped into eight categories. The {rs} is a placeholder representing an rs number.

To ensure valid records from the TogoVar API, we selected 30 rs numbers cited in recent publications (2023 onward) using the mutation2pubtator3 file of PubTator3. For manual evaluation, reflecting real-world usage, we constructed 1500 questions by combining 50 templates with 30 rs numbers, then randomly selected 150 questions under the constraint that each template appeared at least once.

Two healthcare professionals manually evaluated 450 answers (150 questions × three systems: ChatTogoVar, GPT-4o and VarChat). The primary rater specialized in phenotype–genotype relationships, and the secondary in genomic variant database design. Each scored answers on five criteria: accuracy, completeness, logical consistency, clarity and conciseness, and evidence support using a 0–10 scale. The total score (range 0–50) was defined as the sum of these criteria. For analysis, we used the mean of both raters’ scores per criterion and the total score.

### LLM-based evaluation

Although the 150 manually evaluated questions provided a strong foundation, the limited sample size constrained statistical power. Therefore, for evaluation with GPT-4o, we used 1500 template–rs-number combinations that had been constructed in advance, thereby increasing variant diversity and statistical power. The evaluation process, including the design of the evaluation prompt (Supplementary Fig. 3), followed the same procedure and criteria as the manual evaluation. This setting enabled us to obtain robust and consistent evaluation results that complemented the manual evaluation.

### Statistical analysis

Manual evaluation scores were independently assigned by two raters, and the mean of the two raters’ scores was used for the primary analysis. For each evaluation setting, we report mean scores with 95% bootstrap confidence intervals (CIs). Overall differences among the three systems were assessed using the Friedman test (paired across systems within the same questions), and Kendall’s *W* was reported as an effect size (Tables 4 and 5). In addition, we performed pairwise comparisons using the paired Wilcoxon signed-rank test with Holm adjustment for multiple comparisons (Supplementary Tables 2 and 3).

Inter-rater agreement for manual total scores, based on the mean of the two raters’ scores, was quantified using ICC(2,2)^23^ (intraclass correlation coefficient; two-way random-effects, absolute agreement) and quadratic-weighted Cohen’s *κ*, and absolute differences between raters’ total scores were summarized (Supplementary Table 4).

### Environment setup

Environment setup and secret handling follow standard Azure OpenAI practices. To reproduce the answer-generation and evaluation pipelines, users copy .env.template and set deployment-specific values (for example, api_key, api_base/endpoint and deployment_name). Secrets are managed via environment variables and not stored in the repository. Procedures for pipeline reproduction and public demo setup are documented in the ChatTogoVar project repository^24^. For cost control, we refer readers to Azure pricing^25^, Cost Management budgets/alerts^26^ and Azure OpenAI quotas/limits^27^.

### Development of a public website powered by a self-hosted LLM

To allow users to experience ChatTogoVar, we developed a website powered by a self-hosted LLM (Qwen3-30B-A3B-Instruct-2507-GGUF^28^). The server is configured with a 262,144-token context window (--ctx-size 262144) and runs on two NVIDIA Tesla V100 graphics processing units (32 GB memory each) with layer-wise model parallelism across two graphics processing units. The website is available at https://chattogovar.dbcls.jp (Supplementary Fig. 2). The deployed backend (Qwen3-30B-A3B-Instruct-2507-GGUF) is described as a multilingual model (including Japanese) enabling responses in Japanese when queries are submitted in Japanese or explicitly request Japanese output.

## Results

In April 2025, we conducted both manual and LLM-based evaluations. We present the results of these evaluations and compare the answers to an example question.

### Manual evaluation

Among the 150 questions selected for manual evaluation, the highest-scoring answers were 135 (90.0%) for ChatTogoVar, 3 (2.0%) for GPT-4o, and 10 (6.7%) for VarChat, with 2 ties and no unique winner (1.3%) (Table 3). Across the five evaluation criteria (accuracy, completeness, logical consistency, clarity and conciseness, and evidence support), ChatTogoVar achieved the highest mean scores. Differences among the three systems were significant for each criterion (Friedman test, *P* < 0.001), with large effect sizes (Kendall’s *W* = 0.587–0.738) (Table 4). GPT-4o showed particularly low mean scores for accuracy (2.35) and evidence support (2.76) (Table 4). Post-hoc Wilcoxon signed-rank tests with Holm correction (Supplementary Table 2) showed that ChatTogoVar scored significantly higher than both GPT-4o and VarChat for all five criteria (*P* < 0.001).

**Table 3:** The number of highest-scoring answers per system.

System	Manual evaluation	LLM-based evaluation
ChatTogoVar	135 (90.0%)	1395 (93.0%)
GPT-4o	3 (2.0%)	91 (6.1%)
VarChat	10 (6.7%)	12 (0.8%)
Tie (no unique winner)	2 (1.3%)	2 (0.1%)
Total	150 (100.0%)	1500 (100.0%)

Results are shown for the manual evaluation (150 questions) and the LLM-based evaluation (1500 questions). For the manual evaluation, we report the mean of the two raters’ total scores.

**Table 4:** Comparison of mean total and criterion scores among ChatTogoVar, GPT-4o and VarChat in manual (*n* = 150) and LLM-based (*n* = 1500) evaluation settings.

Evaluation setting	Evaluation criterion	ChatTogoVar	GPT-4o	VarChat	Friedman *P* value	Kendall’s *W*	Questions
Manual (mean)	Total	40.68 (39.92–41.42)	22.30 (21.13–23.49)	30.70 (29.71–31.76)	<0.001	0.693	150
Manual (mean)	Accuracy	7.94 (7.75–8.12)	2.35 (1.98–2.73)	5.16 (4.83–5.50)	<0.001	0.688	150
Manual (mean)	Completeness	7.16 (6.88–7.43)	3.62 (3.33–3.92)	3.81 (3.43–4.22)	<0.001	0.59	150
Manual (mean)	Logical consistency	8.69 (8.55–8.83)	6.62 (6.45–6.80)	7.51 (7.35–7.68)	<0.001	0.587	150
Manual (mean)	Clarity and conciseness	8.61 (8.49–8.73)	6.95 (6.80–7.10)	6.52 (6.36–6.68)	<0.001	0.644	150
Manual (mean)	Evidence support	8.28 (8.13–8.43)	2.76 (2.38–3.14)	7.69 (7.52–7.85)	<0.001	0.738	150
LLM	Total	44.53 (44.35–44.71)	31.98 (31.70–32.26)	29.63 (29.17–30.10)	<0.001	0.653	1500
LLM	Accuracy	9.51 (9.46–9.56)	5.92 (5.84–6.00)	6.09 (5.97–6.20)	<0.001	0.632	1500
LLM	Completeness	9.05 (9.00–9.09)	6.44 (6.38–6.50)	5.90 (5.79–6.00)	<0.001	0.646	1500
LLM	Logical consistency	9.68 (9.64–9.71)	7.26 (7.20–7.32)	6.93 (6.82–7.03)	<0.001	0.589	1500
LLM	Clarity and conciseness	8.88 (8.85–8.91)	7.12 (7.07–7.18)	6.01 (5.92–6.09)	<0.001	0.697	1500
LLM	Evidence support	7.42 (7.36–7.48)	5.25 (5.17–5.32)	4.87 (4.78–4.97)	<0.001	0.574	1500

Mean scores are reported for the total score and five evaluation criteria (accuracy, completeness, logical consistency, clarity and conciseness, and evidence support). Values are means with 95% bootstrap CIs. For the manual evaluation, values are based on the mean of the two raters’ scores. Overall differences among the three systems were assessed using the Friedman test, with scores paired within questions, and Kendall’s *W* was reported as an effect size. Pairwise post-hoc comparisons are provided in Supplementary Table 2.

When aggregated by question category, ChatTogoVar achieved the largest number of highest-scoring answers and the highest mean Total scores in all eight categories (Table 5). Overall system differences within each question category were consistently significant (Friedman *P* < 0.001) with large effect sizes (Kendall’s *W* = 0.586–1) (Table 5). Post-hoc Wilcoxon signed-rank tests with Holm adjustment (Supplementary Table 3) again showed ChatTogoVar scoring higher than both comparators in all categories, with median paired differences (ChatTogoVar − comparator) ranging from +6.5 to +23.5 in total score.

**Table 5:** Comparison of question-category–level mean total scores among ChatTogoVar, GPT-4o and VarChat in the manual (*n* = 150) and LLM-based (*n* = 1500) evaluation settings.

Evaluation setting	Question category	Number of highest-scoring answers				Mean total scores				
		ChatTogoVar	GPT-4o	VarChat	Ties	ChatTogoVar	GPT-4o	VarChat	Friedman *P* value	Kendall’s *W*
Manual (mean)	Basic information	23	0	0	0	43.43 (41.50–45.35)	18.59 (15.15–22.28)	32.85 (29.83–35.93)	<0.001	0.852
Manual (mean)	Allele frequency and population distribution	19	1	0	1	42.17 (41.17–43.17)	26.64 (23.67–29.95)	24.81 (23.38, 26.31)	<0.001	0.679
Manual (mean)	Clinical significance	15	1	6	1	42.54 (40.98–44.00)	21.83 (19.65–24.22)	37.41 (34.65–40.26)	<0.001	0.586
Manual (mean)	Pharmacogenomics	15	0	1	0	37.97 (35.75–40.09)	22.00 (18.50–26.03)	28.81 (27.25–30.53)	<0.001	0.769
Manual (mean)	Functional impact	23	0	2	0	41.22 (39.82–42.52)	21.60 (19.56–23.78)	33.02 (30.76–35.38)	<0.001	0.779
Manual (mean)	Evolutionary context	13	0	0	0	40.42 (39.00–41.69)	20.27 (17.92–23.12)	31.04 (29.50–32.38)	<0.001	1
Manual (mean)	Comparison with related variants	16	1	1	0	33.39 (31.78–35.00)	21.39 (19.22–23.75)	27.06 (25.94–28.19)	<0.001	0.67
Manual (mean)	Databases and Bioinformatics analysis	11	0	0	0	43.09 (41.82–44.55)	28.73 (23.45–33.91)	26.41 (25.32–27.55)	<0.001	0.776
LLM	Basic information	227	11	0	2	45.84 (45.23–46.38)	30.32 (29.43–31.23)	25.71 (24.28–27.19)	<0.001	0.717
LLM	Allele frequency and population distribution	172	38	0	0	42.44 (41.85–43.02)	35.78 (35.00–36.57)	21.34 (20.50–22.17)	<0.001	0.793
LLM	Clinical significance	213	17	10	0	44.77 (44.33–45.20)	32.54 (31.91–33.17)	35.15 (34.22–36.09)	<0.001	0.587
LLM	Pharmacogenomics	137	12	1	0	43.87 (43.28–44.37)	32.89 (32.01–33.81)	29.72 (28.75–30.70)	<0.001	0.649
LLM	Functional impact	207	3	0	0	45.60 (45.30–45.89)	30.66 (30.19–31.12)	35.44 (34.59–36.30)	<0.001	0.782
LLM	Evolutionary context	147	3	0	0	44.73 (44.31–45.08)	30.53 (30.03–31.07)	31.20 (29.75–32.54)	<0.001	0.73
LLM	Comparison with related variants	179	0	1	0	44.07 (43.68–44.42)	29.32 (28.81–29.81)	31.73 (30.78–32.68)	<0.001	0.753
LLM	Databases and bioinformatics analysis	113	7	0	0	44.53 (43.85–45.12)	34.57 (33.73–35.42)	25.57 (24.32–26.84)	<0.001	0.871

Values are means with 95% bootstrap CIs; for the manual evaluation, values are based on the mean of the two raters’ scores. Friedman *P* values and Kendall’s *W* are reported to summarize overall differences among the three systems within each evaluation setting × question category. ‘Number of highest-scoring answers’ counts questions for which a system achieved a unique highest total score, and ‘Tie (no unique winner)’ counts questions for which the highest total score was shared by two or more systems (ties are not attributed to any single system). Pairwise comparisons are provided in Supplementary Table 3.

### Reproducibility of the manual evaluation

Inter-rater agreement for manual total scores is summarized in Supplementary Table 4. We quantified inter-rater agreement using ICC(2,2), the average-measures ICC corresponding to the reliability of the mean of two raters’ scores. Pooled across all systems (*n* = 450 system–question instances), agreement was good (ICC(2,2) of 0.814), with a median absolute rater difference of 5 points (mean 6.629; max 28). System-stratified analyses showed heterogeneity in agreement (Supplementary Table 4), with ICC(2,2) ranging from 0.361 to 0.660 across systems, consistent with the use of averaged scores for the main manual analyses. Consistently, rater-stratified winner counts based on total scores showed that ChatTogoVar was most frequently ranked as the unique highest-scoring system by both raters (Supplementary Table 1).

### LLM-based evaluation

In the evaluation of 1,500 questions by GPT-4o, the distribution of highest-scoring answers was 1,395 (93.0%) for ChatTogoVar, 91 (6.1%) for GPT-4o, 12 (0.8%) for VarChat, and 2 ties with no unique winner (0.1%) (Table 3). Compared with the manual evaluation, GPT-4o surpassed VarChat in this setting. For the mean scores of each criterion, GPT-4o achieved 5.92 for accuracy and 5.25 for evidence support, which were higher than its scores in the manual evaluation (2.35 and 2.76, respectively) (Table 4).

### Case study of differences in variant interpretation among ChatTogoVar, GPT-4o and VarChat

We compared the answers of ChatTogoVar, GPT-4o and VarChat to the question ‘What is the clinical significance of rs34637584 in ClinVar?’ (Supplementary Fig. 4). The correct answer is that rs34637584 is pathogenic for several diseases, including autosomal dominant Parkinson disease 8, as described in the ClinVar entry VCV000001940.

The answer generated by ChatTogoVar correctly presented the pathogenicity and conditions listed in VCV000001940 and explicitly cited this entry as supporting evidence. VarChat’s answer also included the statement “This variant has been widely associated with Parkinson’s disease (PD) and is known to increase kinase activity of LRRK2, which is implicated in the pathogenesis of the disease”, thereby correctly identifying the associated disease and gene. It also included the relevant ClinVar entry number. However, the response contained irrelevant information not directly related to the question, which reduced clarity and conciseness in the LLM-based evaluation.

Conversely, GPT-4o’s answer mistakenly identified the gene containing rs34637584 as phosphatase and tensin homolog (PTEN) and consequently reported an incorrect association with PTEN hamartoma tumor syndrome. Similar errors were observed in other questions, where GPT-4o failed to correctly identify the gene harboring the target variant and produced responses based on an incorrect variant–gene relationship.

These differences were also reflected in the manual and LLM-based evaluation scores (Supplementary Table 5). ChatTogoVar achieved high mean total scores of 45–47 out of 50 in both evaluations. By contrast, GPT-4o scored as low as 1 point in accuracy and evidence support during the manual evaluation, while VarChat showed a low score of 3 points in clarity and conciseness during the LLM-based evaluation.

## Discussion

ChatTogoVar achieved the highest overall scores by retrieving accurate and reliable information from TogoVar through the RAG framework. Using variant identifiers, it effectively integrated key attributes, such as gene symbols, allele frequencies and clinical significance from trusted databases, including ClinVar and gnomAD. The evaluation, combining manual and LLM-based assessments across diverse question categories, demonstrated that grounding LLMs in TogoVar reduced hallucinations in variant interpretation. Although recent advances in general-purpose LLMs have significantly improved their capacity to address biomedical questions, their knowledge remains inherently constrained by static training data. In genomics, continuous updates to variant frequency data and clinical interpretations—often highly population-specific—are essential for accurate variant assessment. Our results also indicate that general-purpose LLMs may still yield incorrect gene–variant associations or unsupported claims in this context. Thus, systems, such as ChatTogoVar, provide a complementary approach by grounding responses in up-to-date, population-specific variant information, particularly when hallucination risks must be minimized and timely interpretation is required.

Quantitative results (Tables 3–5) were obtained using the GPT-4o-backed configuration of ChatTogoVar. The public website, however, operates with a self-hosted open-weight model for cost and operational reasons, and its responses may differ. We used GPT-4o for large-scale answer generation and automated evaluation to ensure practical throughput and stable execution, as on-premise servers are slower for high-volume queries. While some open-weight models (for example, Qwen3-30B-A3B-Instruct-2507) can be competitive with GPT-4o on some benchmarks^28,29^, the choice of backend LLM in practice reflects operational constraints as well as model characteristics.

In this study, we intentionally limited the question format to those containing only a single rs number or HGVS notation to simplify API queries and ensure controlled evaluation conditions. This design enabled us to isolate and clearly demonstrate the direct contribution of TogoVar-based retrieval. However, it does not yet cover more complex, real-world scenarios involving multiple variants, phenotype-driven search tasks or exploratory genomic queries. Emerging frameworks, such as the Model Context Protocol^30^ could allow LLMs to automatically translate natural-language questions into structured TogoVar API calls, enabling multivariant analysis and population-specific filtering (for example, allele frequency thresholds, linkage disequilibrium-based variant grouping and haplotype retrieval). ChatTogoVar also exhibited some limitations in areas not yet fully covered by TogoVar, such as ‘pharmacogenomics and drug metabolism’ and ‘comparison with related variants’ (Table 5). Structural-variant queries also remain outside its scope, because structural-variant annotations are absent from the current TogoVar release. These limitations could be mitigated by dynamically using external databases through their APIs to add background knowledge when needed. To enhance pharmacogenomics and drug metabolism data, the PharmGKB^31^ and the Clinical Pharmacogenetics Implementation Consortium^32^ databases should be incorporated into TogoVar. To strengthen the relationships between variants, we should utilize Japanese haplotypes available in the Joint Open Genome and Omics Platform (JoGo) database^33^. The development of a Model Context Protocol-based ecosystem would make such extensions more efficient and allow better integration of additional data sources.

Our evaluation used GPT-4o both as the system under test and as the LLM-based evaluator, which may introduce self-evaluation bias in the automated assessment. Importantly, however, the main conclusions are supported by an independent manual evaluation by two healthcare professionals, which consistently confirmed ChatTogoVar’s superiority across all criteria and categories. Inter-rater reliability analyses showed good agreement and consistent conclusions across raters (Supplementary Table 4), supporting the robustness of the manual findings. Rater-specific analyses likewise yielded consistent conclusions (Supplementary Tables 2-2, 2-3, 3-2 and 3-3), justifying the use of the mean of the two raters’ scores in the main manual analyses.

In the future, we plan to enhance both the usability and reliability of variant interpretation. A new user interface, similar to Google’s AI-powered search mode, will present a concise ChatTogoVar summary at the top of each TogoVar page, followed by the factual variant data currently stored in TogoVar. This design will allow users to quickly grasp the key interpretation generated by ChatTogoVar while maintaining direct access to underlying evidence and database records.

## Supplementary information

Supplementary Information
