# Supplementary material for: ChatTogoVar: a TogoVar-based retrieval-augmented generation system for precise genomic variant interpretation
Source: Hum Genome Var. 2026 Apr 9;13:12. doi: 10.1038/s41439-026-00344-4 (PMC13194818; doi:10.1038/s41439-026-00344-4)
Supplement: Supplementary file 1 — Supplementary Information [file 41439_2026_344_MOESM1_ESM.pdf]

Supplementary Fig. 1. How variant information is retrieved from TogoVar.

The following text shows how variant information of rs34637584 is retrieved from TogoVar.

a. Request query

```
curl -X 'POST' \
  'https://grch38.togovar.org/api/search/variant' \
  -H 'accept: application/json' \
  -H 'Content-Type: application/json' \
  -d '{
  "query": {
    "id": [
      "rs34637584"
    ]
  }
}'
```

b. Response in JSON format returned from TogoVar


```
{
  "scroll": {
    "offset": 0,
    "limit": 100,
    "max_rows": 10000
  },
  "statistics": {
    "total": 1097708150,
    "filtered": 1,
    "dataset": {
      "gem_j_wga": 1,
      "gnomad_exomes": 1,
      "gnomad_genomes": 1,
      "ncbn": 1,
      "tommo": 1,
      "clinvar": 1,
      "mgend": 1,
      "jga_wgs": 0,
      "jga_wes": 0,
      "jga_snp": 0
    },
    "type": {
      "SO_0001483": 1,
      "SO_1000002": 0,
      "SO_0000667": 0,
      "SO_0000159": 0,
      "SO_1000032": 0
    },
    "significance": {
      "P": 5,
      "LP": 1,
      "RF": 1,
      "NC": 0,
      "PLP": 0,
      "LPLP": 0,
      "DR": 0,
      "ERA": 0,
      "LRA": 0,
      "URA": 0,
      "CS": 0,
```

```
      "A": 0,
      "AF": 0,
      "PR": 0,
      "B": 0,
      "LB": 0,
      "CI": 0,
      "AN": 0,
      "O": 0,
      "US": 0,
      "NP": 0
    },
    "consequence": {
      "SO_0001583": 11,
      "SO_0001621": 7,
      "SO_0001624": 6,
      "SO_0001792": 3,
      "SO_0001893": 0,
      "SO_0001574": 0,
      "SO_0001575": 0,
      "SO_0001587": 0,
      "SO_0001589": 0,
      "SO_0001578": 0,
      "SO_0002012": 0,
      "SO_0001889": 0,
      "SO_0001821": 0,
      "SO_0001822": 0,
      "SO_0001818": 0,
      "SO_0001630": 0,
      "SO_0001626": 0,
      "SO_0002019": 0,
      "SO_0001567": 0,
      "SO_0001819": 0,
      "SO_0001580": 0,
      "SO_0001620": 0,
      "SO_0001623": 0,
      "SO_0001627": 0,
      "SO_0001619": 0,
      "SO_0001631": 0,
      "SO_0001632": 0,
      "SO_0001895": 0,
      "SO_0001892": 0,
      "SO_0001782": 0,
      "SO_0001894": 0,
      "SO_0001891": 0,
      "SO_0001907": 0,
      "SO_0001566": 0,
      "SO_0001906": 0,
      "SO_0001628": 0
    }
  },
  "data": [
    {
      "id": "tgv45580587",
      "type": "SO_0001483",
      "chromosome": "12",
      "position": 40340400,
      "reference": "G",
      "alternate": "A",
      "existing_variations": [
        "rs34637584"
      ],
      "symbols": [
        {
          "name": "LRRK2",
```

```
      "id": 18618,
      "synonyms": [
        "ROCO2",
        "DKFZp434H2111",
        "FLJ45829",
        "RIPK7"
      ]
    }
  ],
  "external_link": {
    "dbsnp": [
      {
        "title": "rs34637584",
        "xref": "https://identifiers.org/dbsnp/rs34637584"
      }
    ],
    "clinvar": [
      {
        "title": "VCV000001940",
        "xref": "https://www.ncbi.nlm.nih.gov/clinvar/variation/1940"
      }
    ],
    "mgend": [
      {
        "title": "SNV|hg38|chr12:40340400-40340400|gG>A",
        "xref": "https://mgend.ncgm.go.jp/variant/info/SNV|hg38|chr12:40340400-40340400|gG>A"
      }
    ],
    "tommo": [
      {
        "title": "12:40340400",
        "xref": "https://jmorp.megabank.tohoku.ac.jp/search?query=12%3A40340400"
      }
    ],
    "gnomad": [
      {
        "title": "12-40340400-G-A",
        "xref": "https://gnomad.broadinstitute.org/variant/12-40340400-G-A?dataset=gnomad_r4"
      }
    ]
  },
  "significance": [
    {
      "conditions": [
        {
          "name": "Autosomal dominant Parkinson disease 8",
          "medgen": "C1846862"
        }
      ],
      "interpretations": [
        "P",
        "LP"
      ],
      "submission_count": 14,
      "source": "clinvar"
    },
    {
      "conditions": [
        {
          "name": "not provided",
          "medgen": "C3661900"
        }
      ],
      "interpretations": [
```

```
      "P"
    ],
    "submission_count": 6,
    "source": "clinvar"
  },
  {
    "conditions": [
      {
        "name": "Inborn genetic diseases",
        "medgen": "C0950123"
      }
    ],
    "interpretations": [
      "P"
    ],
    "submission_count": 1,
    "source": "clinvar"
  },
  {
    "conditions": [
      {
        "name": "Parkinson disease, late-onset",
        "medgen": "C3160718"
      }
    ],
    "interpretations": [
      "P"
    ],
    "submission_count": 1,
    "source": "clinvar"
  },
  {
    "conditions": [
      {
        "name": "Parkinson disease",
        "medgen": "C0030567"
      }
    ],
    "interpretations": [
      "P"
    ],
    "submission_count": 1,
    "source": "clinvar"
  },
  {
    "conditions": [
      {
        "name": "Young-onset Parkinson disease",
        "medgen": "C4275179"
      }
    ],
    "interpretations": [
      "RF"
    ],
    "submission_count": 1,
    "source": "clinvar"
  },
  {
    "conditions": [],
    "interpretations": [],
    "submission_count": null,
    "source": "mgend"
  }
],
```

```
"most_severe_consequence": "SO_0001583",
"sift": 0,
"polyphen": 1,
"alphamissense": 0.936,
"transcripts": [
 {
  "hgnc_id": 18618,
  "symbol": {
   "source": "HGNC",
   "label": "LRRK2"
  },
  "sift": 0,
  "transcript_id": "ENST00000298910",
  "consequence": [
   "SO_0001583"
  ],
  "consequence_type": "transcript_consequence",
  "hgvs_p": "ENSP00000298910.7:p.Gly2019Ser",
  "hgvs_c": "ENST00000298910.12:c.6055G>A",
  "alphamissense": 0.936,
  "gene_id": "ENSG00000188906",
  "polyphen": 1,
  "hgvs_g": "chr12:g.40340400G>A"
 },
 {
  "hgnc_id": 18618,
  "symbol": {
   "source": "HGNC",
   "label": "LRRK2"
  },
  "transcript_id": "ENST00000430804",
  "consequence": [
   "SO_0001624",
   "SO_0001621"
  ],
  "consequence_type": "transcript_consequence",
  "hgvs_c": "ENST00000430804.5:c.*2728G>A",
  "gene_id": "ENSG00000188906",
  "hgvs_g": "chr12:g.40340400G>A"
 },
 {
  "hgnc_id": 18618,
  "symbol": {
   "source": "HGNC",
   "label": "LRRK2"
  },
  "transcript_id": "ENST00000479187",
  "consequence": [
   "SO_0001792"
  ],
  "consequence_type": "transcript_consequence",
  "hgvs_c": "ENST00000479187.5:n.2736G>A",
  "gene_id": "ENSG00000188906",
  "hgvs_g": "chr12:g.40340400G>A"
 },
 {
  "hgnc_id": 18618,
  "symbol": {
   "source": "HGNC",
   "label": "LRRK2"
  },
  "transcript_id": "ENST00000679360",
  "consequence": [
   "SO_0001624",
```

```
      "SO_0001621"
    ],
    "consequence_type": "transcript_consequence",
    "hgvs_c": "ENST00000679360.1:c.*4964G>A",
    "gene_id": "ENSG00000188906",
    "hgvs_g": "chr12:g.40340400G>A"
  },
  {
    "hgnc_id": 18618,
    "symbol": {
      "source": "HGNC",
      "label": "LRRK2"
    },
    "transcript_id": "ENST00000679532",
    "consequence": [
      "SO_0001624",
      "SO_0001621"
    ],
    "consequence_type": "transcript_consequence",
    "hgvs_c": "ENST00000679532.1:c.*1315G>A",
    "gene_id": "ENSG00000188906",
    "hgvs_g": "chr12:g.40340400G>A"
  },
  {
    "hgnc_id": 18618,
    "symbol": {
      "source": "HGNC",
      "label": "LRRK2"
    },
    "transcript_id": "ENST00000680018",
    "consequence": [
      "SO_0001624",
      "SO_0001621"
    ],
    "consequence_type": "transcript_consequence",
    "hgvs_c": "ENST00000680018.1:c.*1278G>A",
    "gene_id": "ENSG00000188906",
    "hgvs_g": "chr12:g.40340400G>A"
  },
  {
    "hgnc_id": 18618,
    "symbol": {
      "source": "HGNC",
      "label": "LRRK2"
    },
    "sift": 0,
    "transcript_id": "ENST00000680422",
    "consequence": [
      "SO_0001583",
      "SO_0001621"
    ],
    "consequence_type": "transcript_consequence",
    "hgvs_p": "ENSP00000505304.1:p.Gly568Ser",
    "hgvs_c": "ENST00000680422.1:c.1702G>A",
    "gene_id": "ENSG00000188906",
    "polyphen": 0.998,
    "hgvs_g": "chr12:g.40340400G>A"
  },
  {
    "hgnc_id": 18618,
    "symbol": {
      "source": "HGNC",
      "label": "LRRK2"
    },
```

"transcript_id": "ENST00000680425",
"consequence": [
"SO_0001624",
"SO_0001621"
],
"consequence_type": "transcript_consequence",
"hgvs_c": "ENST00000680425.1:c.*1090G>A",
"gene_id": "ENSG00000188906",
"hgvs_g": "chr12:g.40340400G>A"
},
{
"hgnc_id": 18618,
"symbol": {
"source": "HGNC",
"label": "LRRK2"
},
"transcript_id": "ENST00000680453",
"consequence": [
"SO_0001624",
"SO_0001621"
],
"consequence_type": "transcript_consequence",
"hgvs_c": "ENST00000680453.1:c.*1031G>A",
"gene_id": "ENSG00000188906",
"hgvs_g": "chr12:g.40340400G>A"
},
{
"hgnc_id": 18618,
"symbol": {
"source": "HGNC",
"label": "LRRK2"
},
"sift": 0,
"transcript_id": "ENST00000680790",
"consequence": [
"SO_0001583"
],
"consequence_type": "transcript_consequence",
"hgvs_p": "ENSP00000505335.1:p.Gly1934Ser",
"hgvs_c": "ENST00000680790.1:c.5800G>A",
"gene_id": "ENSG00000188906",
"polyphen": 0.999,
"hgvs_g": "chr12:g.40340400G>A"
},
{
"hgnc_id": 18618,
"symbol": {
"source": "HGNC",
"label": "LRRK2"
},
"transcript_id": "ENST00000681136",
"consequence": [
"SO_0001792"
],
"consequence_type": "transcript_consequence",
"hgvs_c": "ENST00000681136.1:n.2039G>A",
"gene_id": "ENSG00000188906",
"hgvs_g": "chr12:g.40340400G>A"
},
{
"hgnc_id": 18618,
"symbol": {
"source": "HGNC",
"label": "LRRK2"

```
    },
    "sift": 0,
    "transcript_id": "ENST00000681696",
    "consequence": [
     "SO_0001583"
    ],
    "consequence_type": "transcript_consequence",
    "hgvs_p": "ENSP00000505871.1:p.Gly580Ser",
    "hgvs_c": "ENST00000681696.1:c.1738G>A",
    "gene_id": "ENSG00000188906",
    "polyphen": 0.998,
    "hgvs_g": "chr12:g.40340400G>A"
   },
   {
    "hgnc_id": 18618,
    "symbol": {
     "source": "EntrezGene",
     "label": "LRRK2"
    },
    "sift": 0,
    "transcript_id": "NM_198578.4",
    "consequence": [
     "SO_0001583"
    ],
    "consequence_type": "transcript_consequence",
    "hgvs_p": "NP_940980.4:p.Gly2019Ser",
    "hgvs_c": "NM_198578.4:c.6055G>A",
    "alphamissense": 0.936,
    "gene_id": "120892",
    "polyphen": 1,
    "hgvs_g": "chr12:g.40340400G>A"
   },
   {
    "hgnc_id": 18618,
    "symbol": {
     "source": "EntrezGene",
     "label": "LRRK2"
    },
    "transcript_id": "XM_005268629.5",
    "consequence": [
     "SO_0001583"
    ],
    "consequence_type": "transcript_consequence",
    "hgvs_p": "XP_005268686.1:p.Gly2019Ser",
    "hgvs_c": "XM_005268629.5:c.6055G>A",
    "alphamissense": 0.936,
    "gene_id": "120892",
    "hgvs_g": "chr12:g.40340400G>A"
   },
   {
    "hgnc_id": 18618,
    "symbol": {
     "source": "EntrezGene",
     "label": "LRRK2"
    },
    "transcript_id": "XM_011537877.4",
    "consequence": [
     "SO_0001583"
    ],
    "consequence_type": "transcript_consequence",
    "hgvs_p": "XP_011536179.1:p.Gly2019Ser",
    "hgvs_c": "XM_011537877.4:c.6055G>A",
    "alphamissense": 0.936,
    "gene_id": "120892",
```

```
    "hgvs_g": "chr12:g.40340400G>A"
  },
  {
    "hgnc_id": 18618,
    "symbol": {
      "source": "EntrezGene",
      "label": "LRRK2"
    },
    "transcript_id": "XM_017018787.2",
    "consequence": [
      "SO_0001583"
    ],
    "consequence_type": "transcript_consequence",
    "hgvs_p": "XP_016874276.1:p.Gly991Ser",
    "hgvs_c": "XM_017018787.2:c.2971G>A",
    "gene_id": "120892",
    "hgvs_g": "chr12:g.40340400G>A"
  },
  {
    "hgnc_id": 18618,
    "symbol": {
      "source": "EntrezGene",
      "label": "LRRK2"
    },
    "transcript_id": "XM_024448833.2",
    "consequence": [
      "SO_0001583"
    ],
    "consequence_type": "transcript_consequence",
    "hgvs_p": "XP_024304601.1:p.Gly1618Ser",
    "hgvs_c": "XM_024448833.2:c.4852G>A",
    "gene_id": "120892",
    "hgvs_g": "chr12:g.40340400G>A"
  },
  {
    "hgnc_id": 18618,
    "symbol": {
      "source": "EntrezGene",
      "label": "LRRK2"
    },
    "transcript_id": "XM_047428277.1",
    "consequence": [
      "SO_0001583"
    ],
    "consequence_type": "transcript_consequence",
    "hgvs_p": "XP_047284233.1:p.Gly2019Ser",
    "hgvs_c": "XM_047428277.1:c.6055G>A",
    "alphamissense": 0.936,
    "gene_id": "120892",
    "hgvs_g": "chr12:g.40340400G>A"
  },
  {
    "hgnc_id": 18618,
    "symbol": {
      "source": "EntrezGene",
      "label": "LRRK2"
    },
    "transcript_id": "XM_047428278.1",
    "consequence": [
      "SO_0001583"
    ],
    "consequence_type": "transcript_consequence",
    "hgvs_p": "XP_047284234.1:p.Gly2019Ser",
    "hgvs_c": "XM_047428278.1:c.6055G>A",
```

```
      "alphamissense": 0.936,
      "gene_id": "120892",
      "hgvs_g": "chr12:g.40340400G>A"
    },
    {
      "hgnc_id": 18618,
      "symbol": {
        "source": "EntrezGene",
        "label": "LRRK2"
      },
      "transcript_id": "XR_007063041.1",
      "consequence": [
        "SO_0001792"
      ],
      "consequence_type": "transcript_consequence",
      "hgvs_c": "XR_007063041.1:n.6190G>A",
      "gene_id": "120892",
      "hgvs_g": "chr12:g.40340400G>A"
    }
  ],
  "frequencies": [
    {
      "ac": 3,
      "af": 0.000028000000384054147,
      "an": 108588,
      "filter": [
        "PASS"
      ],
      "quality": 1703.5899658203125,
      "source": "tommo"
    },
    {
      "ac": 609,
      "af": 0.00041666001197881997,
      "an": 1461622,
      "filter": [
        "PASS"
      ],
      "source": "gnomad_exomes"
    },
    {
      "ac": 55,
      "af": 0.00036140900920145214,
      "an": 152182,
      "filter": [
        "PASS"
      ],
      "source": "gnomad_genomes"
    },
    {
      "ac": 1,
      "af": 0.00006589999975403771,
      "an": 15174,
      "filter": [
        "PASS"
      ],
      "quality": 380.29998779296875,
      "source": "gem_j_wga"
    },
    {
      "aac": 0,
      "ac": 3,
      "af": 0.00012744299601763487,
      "an": 23540,
```

```
          "filter": [
            "PASS"
          ],
          "quality": 3056.469970703125,
          "source": "ncbn"
        }
      ]
    }
  ]
}
```

Supplementary Fig. 2. ChatTogoVar Public Website Powered by a Self-Hosted LLM

ChatTogoVar is publicly accessible at https://chattogovar.dbcls.jp. The web application is implemented using the Dify framework and is backed by a self-hosted open-weight LLM. In the current deployment, Qwen3-30B-A3B-Instruct-2507 is served via llama.cpp as a GGUF model (Q5_K_M) obtained from Hugging Face. The service supports Japanese queries and can return Japanese responses.

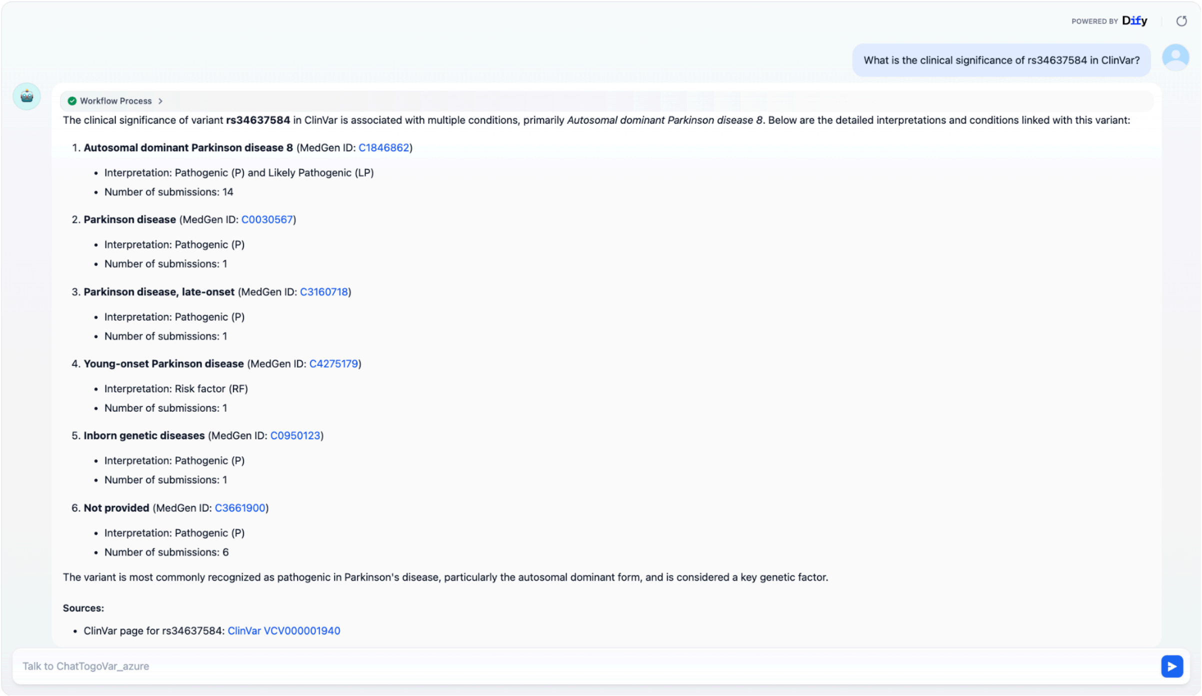

Supplementary Fig. 3. GPT-4o prompt used to evaluate answers generated by ChatTogoVar, GPT-4o, and VarChat.

Variables enclosed in parentheses are filled with values when calling the GPT-4o API. The placeholder {question} is a question statement including one rs number. {chat_togovar}, {chat_gpt}, and {varchat} are answers from ChatTogoVar, GPT-4o, and VarChat.

—-------

Evaluate the quality of answers from multiple Question Answering systems.

Input format:

Questions and answers are provided in the following format:

Question:

{question}

Answer 1: ChatTogoVar

{chat_togovar}

Answer2: GPT-4o

{chat_gpt}

Answer3: VarChat

{varchat}

Gold standard

Gene symbols: {gene_symbols}

Evaluation Criteria:

1. Accuracy: How accurately does the response address the question? Point out any inaccuracies or missing information. Please refer to the gene symbols in the 'Gold standard' above as the correct genes in which the variant mentioned in the question is located.
2. Completeness: Does the response itself provide all the necessary information required to fully answer the question, rather than just pointing to potential sources? Highlight any gaps where specific details should have been provided.
3. Logical Consistency: Does the response maintain logical coherence, with no contradictions?

4. Clarity and Conciseness: Is the response clear and concise, with no ambiguity? Identify any overly verbose or unclear sections.
5. Evidence Support: Does the response rely on credible sources or evidence? Note if the support provided is insufficient or untrustworthy.

Instructions:

Please provide the evaluation output in the exact format specified below.

- Follow the exact structure and formatting of the "Output Format" section.
- Do not add or remove any symbols (e.g., colons, bullet points, spaces).
- Ensure the alignment and indentation are consistent with the "Output Format."
- Replace "X" with the appropriate value or text based on the evaluation.
- If "Reason" is not applicable, write "Not provided" but keep the "Reason" line in the output.
- Every reason should be written in both English and Japanese.

Only output in this format. Do not include additional text or explanations outside of the format.

Output Format:

Question

{question}

Final Result

- Best Answer: X
- Total Score for ChatTogoVar: X/50
- Total Score for GPT-4o: X/50
- Total Score for VarChat: X/50
- Reason:
  - English: X
  - Japanese: X

Scores by Evaluation Criteria

Answer ChatTogoVar
- Accuracy Score: X/10
  - Reason:
    - English: X
    - Japanese: X
- Completeness Score: X/10
  - Reason:
    - English: X
    - Japanese: X

- Logical Consistency Score: X/10
  - Reason:
    - English: X
    - Japanese: X
- Clarity and Conciseness Score: X/10
  - Reason:
    - English: X
    - Japanese: X
- Evidence Support Score: X/10
  - Reason:
    - English: X
    - Japanese: X
- Total Score: X/50

Answer GPT-4o
- Accuracy Score: X/10
  - Reason:
    - English: X
    - Japanese: X
- Completeness Score: X/10
  - Reason:
    - English: X
    - Japanese: X
- Logical Consistency Score: X/10
  - Reason:
    - English: X
    - Japanese: X
- Clarity and Conciseness Score: X/10
  - Reason:
    - English: X
    - Japanese: X
- Evidence Support Score: X/10
  - Reason:
    - English: X
    - Japanese: X
- Total Score: X/50

Answer VarChat
- Accuracy Score: X/10
  - Reason:
    - English: X
    - Japanese: X
- Completeness Score: X/10
  - Reason:

- English: X
- Japanese: X
- Logical Consistency Score: X/10
  - Reason:
    - English: X
    - Japanese: X
- Clarity and Conciseness Score: X/10
  - Reason:
    - English: X
    - Japanese: X
- Evidence Support Score: X/10
  - Reason:
    - English: X
    - Japanese: X
- Total Score: X/50

Supplementary Fig. 4. Case study: differences in variant interpretation among ChatTogoVar, GPT-4o, and VarChat.

Answers generated by a. ChatTogoVar, b. GPT-4o, and c. VarChat in response to the question “What is the clinical significance of rs34637584 in ClinVar?”.

a. Answer by ChatTogoVar

Clinical Significance of rs34637584 in ClinVar

The variant rs34637584 has been reported in ClinVar with the following clinical significance:

1. Pathogenic (P):
    - Associated with Autosomal dominant Parkinson disease 8 (MedGen ID: C1846862).
    - Associated with Parkinson disease, late-onset (MedGen ID: C3160718).
    - Associated with Parkinson disease (MedGen ID: C0030567).
    - Associated with Inborn genetic diseases (MedGen ID: C0950123).
    - Also reported as "not provided" (MedGen ID: C3661900).
2. Risk Factor (RF):
    - Associated with Young-onset Parkinson disease (MedGen ID: C4275179).
3. Likely Pathogenic (LP):
    - Associated with Autosomal dominant Parkinson disease 8.

These classifications are based on multiple submissions to ClinVar, with a total of 14 submissions for "Pathogenic" and 1 submission for "Risk Factor."

Source:

- ClinVar entry for rs34637584: ClinVar VCV000001940

b. Answer by GPT-4o

The variant rs34637584 is associated with clinical significance in ClinVar. Below is the detailed information:

1. Variant Identification:

- rs Number: rs34637584
- HGVS Notation: NM_000314.6(PTEN):c.1026+1G>A
- Gene Name: PTEN (Phosphatase and Tensin Homolog)
- Transcript Name: NM_000314.6
- Links: ClinVar Entry for rs34637584

2. Disease Associations:

This variant is classified as pathogenic in ClinVar. It is associated with PTEN Hamartoma Tumor Syndrome (PHTS), which includes conditions such as Cowden syndrome and Bannayan-Riley-Ruvalcaba syndrome. These syndromes are characterized by an increased risk of benign and malignant tumors, developmental delay, and macrocephaly.

Predictive models such as SIFT and PolyPhen are not applicable for this splice-site variant, but its pathogenicity is supported by functional studies and clinical observations.

3. Literature Evidence:

Several studies have reported this variant in individuals with PTEN-related syndromes. For example:

- Tan et al., 2007: Identified PTEN mutations, including rs34637584, in patients with Cowden syndrome.
- PMID: 17526800

4. Allele Frequency Comparison:

This variant is extremely rare and has not been observed in large population databases such as gnomAD or 1000 Genomes. Its rarity supports its pathogenic classification.

5. GWAS Findings:

No GWAS studies have directly linked rs34637584 to phenotypes beyond its established association with PTEN Hamartoma Tumor Syndrome.

c. Answer by VarChat

The LRRK2 gene encodes for leucine-rich repeat kinase 2, a serine-threonine protein kinase that belongs to the ROCO protein family and plays a significant role in various cellular processes, including the immune response and autophagy. The c.6055G>A p.Gly2019Ser rs34637584 variant in the LRRK2 gene, commonly referred to as p.G2019S, is a missense mutation that results in the substitution of glycine with serine at position 2019 of the protein. This variant has been widely associated with Parkinson's disease (PD) and is known to increase kinase activity of LRRK2, which is implicated in the pathogenesis of the disease [1][2][3][4][5].The p.G2019S variant is particularly prevalent in certain populations, accounting for over 30% of PD cases in Tunisian Arab-Berber individuals and is also common in Ashkenazi Jews and North African Arab-Berbers. It has been suggested that carriers of this variant are descendants of a common ancestral founder, with the allele estimated to have originated at least 40 generations ago. The variant demonstrates incomplete penetrance and variable expressivity, which indicates that other genetic or environmental factors may modulate the clinical manifestation of PD in carriers [1].In addition to its association with PD, the p.G2019S variant has been linked to an elevated risk of

certain cancers, including colon cancer. Experimental models have shown that the variant promotes pathogenesis of colon cancer, characterized by increased tumor number and size, and enhances intestinal epithelial cell proliferation and inflammation within the tumor microenvironment [2]. At the cellular level, the p.G2019S variant has been shown to induce mitochondrial DNA damage and affect mitochondrial dynamics, which may contribute to the neurodegenerative processes observed in PD. The variant also influences the structure-function relationships in human cortical neural networks, leading to aberrant morphology and heightened metabolic demand [3][5].Furthermore, the LRRK2 p.G2019S variant has been implicated in the immune response, with carriers showing increased levels of pro-inflammatory cytokines. It is hypothesized that the variant's impact on the innate immune system may influence the penetrance of PD, as LRRK2 is highly expressed in immune cells and can be induced upon stimulation with pro-inflammatory signals or pathogen exposure [1].Overall, the c.6055G>A p.Gly2019Ser rs34637584 variant in the LRRK2 gene is a significant genetic factor in PD, with implications for immune system function and potential links to cancer pathogenesis. The variant's high prevalence in specific populations and its impact on kinase activity make it a focal point for understanding the genetic underpinnings of PD and exploring targeted therapeutic interventions [1][2][3][4][5].

References

[1] Guenther DT et al. (2024). "The Evolution of Genetic Variability at the LRRK2 Locus." Genes, 15(7) [PubMed](https://pubmed.ncbi.nlm.nih.gov/39062657/)
[2] Wang Y et al. (2024). "LRRK2 G2019S Promotes Colon Cancer Potentially via LRRK2-GSDMD Axis-Mediated Gut Inflammation." Cells, 13(7) [PubMed](https://pubmed.ncbi.nlm.nih.gov/38607004/)
[3] Pena N et al. (2024). "G2019S selective LRRK2 kinase inhibitor abrogates mitochondrial DNA damage." NPJ Parkinson's disease, 10(1) [PubMed](https://pubmed.ncbi.nlm.nih.gov/38429321/)
[4] Ngo HKC et al. (2024). "Short-term lipopolysaccharide treatment leads to astrocyte activation in LRRK2 G2019S knock-in mice without loss of dopaminergic neurons." Research square, () [PubMed](https://pubmed.ncbi.nlm.nih.gov/38562908/)
[5] Valderhaug VD et al. (2024). "Micro-and mesoscale aspects of neurodegeneration in engineered human neural networks carrying the LRRK2 G2019S mutation." Frontiers in cellular neuroscience, 18() [PubMed](https://pubmed.ncbi.nlm.nih.gov/38644975/)
[6] Wang K et al. (2016). "Efficient Generation of Orthologous Point Mutations in Pigs via CRISPR-assisted ssODN-mediated Homology-directed Repair." Molecular therapy. Nucleic acids, 5(11) [PubMed](https://pubmed.ncbi.nlm.nih.gov/27898095/)
[7] James NG et al. (2012). "Number and brightness analysis of LRRK2 oligomerization in live cells." Biophysical journal, 102(11) [PubMed](https://pubmed.ncbi.nlm.nih.gov/22713584/)
[8] Mestre-Frances N et al. (2018). "Exogenous LRRK2G2019S induces parkinsonian-like pathology in a nonhuman primate." JCI insight, 3(14) [PubMed](https://pubmed.ncbi.nlm.nih.gov/30046008/)
[9] di Caudo C et al. (2020). "CAV-2-Mediated GFP and LRRK2(G2019S) Expression in the Macaca fascicularis Brain." Frontiers in molecular neuroscience, 13() [PubMed](https://pubmed.ncbi.nlm.nih.gov/32269512/)
[10] Sun X et al. (2024). "Comprehensive analysis of clinical and biological features in Parkinson's disease associated with the LRRK2 G2019S mutation: Data from the PPMI

study." Clinical and translational science, 17(1)
[PubMed](https://pubmed.ncbi.nlm.nih.gov/38266062/)
[11] Barreh GA et al. (2024). "The Impact of LRRK2 G2019S on Parkinson's Disease: Clinical Phenotype and Treatment in Tunisian Patients." Journal of movement disorders, 17(3) [PubMed](https://pubmed.ncbi.nlm.nih.gov/38649328/)
[12] Elhadi A et al. (2024). "Multi-method computational evaluation of the inhibitors against leucine-rich repeat kinase 2 G2019S mutant for Parkinson's disease." Molecular diversity, 28(6) [PubMed](https://pubmed.ncbi.nlm.nih.gov/38396210/)
[13] Iovino L et al. (2024). "Investigation of microglial diversity in a LRRK2 G2019S mouse Ry of Parkinson's disease." Neurobiology of disease, 195()
[PubMed](https://pubmed.ncbi.nlm.nih.gov/38527708/)
[14] Kmiecik MJ et al. (2024). "Genetic analysis and natural history of Parkinson's disease due to the LRRK2 G2019S variant." Brain : a journal of neurology, 147(6)
[PubMed](https://pubmed.ncbi.nlm.nih.gov/38804604/)
[15] Guevara CA et al. (2024). "Parkinson's LRRK2-G2019S risk gene mutation drives sex-specific behavioral and cellular adaptations to chronic variable stress." bioRxiv : the preprint server for biology, () [PubMed](https://pubmed.ncbi.nlm.nih.gov/38895277/)

ClinVar Submissions

- [RCV000002017](https://www.ncbi.nlm.nih.gov/clinvar/RCV000002017/): Autosomal dominant Parkinson disease 8
- [RCV000325492](https://www.ncbi.nlm.nih.gov/clinvar/RCV000325492/): not provided
- [RCV000622347](https://www.ncbi.nlm.nih.gov/clinvar/RCV000622347/): Inborn genetic diseases
- [RCV001836691](https://www.ncbi.nlm.nih.gov/clinvar/RCV001836691/): Parkinson disease, late-onset
- [RCV003407254](https://www.ncbi.nlm.nih.gov/clinvar/RCV003407254/): LRRK2-related disorder
- [RCV001195216](https://www.ncbi.nlm.nih.gov/clinvar/RCV001195216/): Young-onset Parkinson disease

Supplementary Table 1. Supplement to Table 3: Number of Questions for Which Each System Achieved the Highest Total Score (by Rater and LLM-Based Evaluation)

This table summarizes, for each evaluation setting, the number and percentage of questions for which each system (ChatTogoVar, GPT-4o, and VarChat) achieved the highest Total score. “Tie (no unique winner)” indicates questions in which the highest Total score was shared by two or more systems; tied cases were not attributed to any single system. Two human raters (primary and secondary) independently evaluated the same set of 150 questions (n = 150), and the LLM-based evaluation was conducted on 1,500 questions (n = 1,500).

System	Primary Rater	Secondary Rater	LLM-Based Evaluation
ChatTogoVar	136 (90.7%)	118 (78.7%)	1,395 (93.0%)
GPT-4o	2 (1.3%)	6 (4.0%)	91 (6.1%)
VarChat	10 (6.7%)	12 (8.0%)	12 (0.8%)
Tie (no unique winner)	2 (1.3%)	14 (9.3%)	2 (0.1%)
Total	150 (100.0%)	150 (100.0%)	1,500 (100.0%)

Supplementary Table 2. Supplement to Table 4: Pairwise Wilcoxon Signed-Rank Tests Between Systems by Evaluation Criterion

Post-hoc pairwise comparisons for Table 4 were performed using two-sided paired Wilcoxon signed-rank tests with Holm-adjusted p-values. Median paired differences were computed as (A − B), where A is the first system and B is the second system listed in the "Comparison" column; positive values indicate higher scores for A than for B.

2-1. Manual (Mean of the primary and secondary raters)

				Wilcoxon Test		
Evaluation Criterion	Comparison	Questions	Median Diff	W	p-value	p-value (Holm)
Total	ChatTogoVar vs GPT-4o	150	18.5	9	<0.001	<0.001
Total	ChatTogoVar vs VarChat	150	10	249	<0.001	<0.001
Total	GPT-4o vs VarChat	150	-8	1538.5	<0.001	<0.001
Accuracy	ChatTogoVar vs GPT-4o	150	6	14.5	<0.001	<0.001
Accuracy	ChatTogoVar vs VarChat	150	3	307.5	<0.001	<0.001
Accuracy	GPT-4o vs VarChat	150	-3.25	1404.5	<0.001	<0.001
Completeness	ChatTogoVar vs GPT-4o	150	3	62.5	<0.001	<0.001
Completeness	ChatTogoVar vs VarChat	150	3.5	354	<0.001	<0.001
Completeness	GPT-4o vs VarChat	150	0	4538.5	0.91	0.91
Logical Consistency	ChatTogoVar vs GPT-4o	150	2	38	<0.001	<0.001
Logical Consistency	ChatTogoVar vs VarChat	150	1	370	<0.001	<0.001
Logical Consistency	GPT-4o vs VarChat	150	-1	1526.5	<0.001	<0.001
Clarity and Conciseness	ChatTogoVar vs GPT-4o	150	1.5	123	<0.001	<0.001

Clarity and Conciseness	ChatTogoVar vs VarChat	150	2	67.5	<0.001	<0.001
Clarity and Conciseness	GPT-4o vs VarChat	150	0.5	2514	<0.001	<0.001
Evidence Support	ChatTogoVar vs GPT-4o	150	6	4	<0.001	<0.001
Evidence Support	ChatTogoVar vs VarChat	150	0.5	1394.5	<0.001	<0.001
Evidence Support	GPT-4o vs VarChat	150	-5.5	12.5	<0.001	<0.001

2-2. Manual (Primary rater)

				Wilcoxon Test		
Evaluation Criterion	Comparison	Questions	Median Diff	W	p-value	p-value (Holm)
Total	ChatTogoVar vs GPT-4o	150	22.5	5.5	<0.001	<0.001
Total	ChatTogoVar vs VarChat	150	8	385.5	<0.001	<0.001
Total	GPT-4o vs VarChat	150	-10	1363	<0.001	<0.001
Accuracy	ChatTogoVar vs GPT-4o	150	7	0	<0.001	<0.001
Accuracy	ChatTogoVar vs VarChat	150	1	297.5	<0.001	<0.001
Accuracy	GPT-4o vs VarChat	150	-6	248	<0.001	<0.001
Completeness	ChatTogoVar vs GPT-4o	150	3	78.5	<0.001	<0.001
Completeness	ChatTogoVar vs VarChat	150	3	380	<0.001	<0.001
Completeness	GPT-4o vs VarChat	150	0	2919.5	0.083	0.083
Logical Consistency	ChatTogoVar vs GPT-4o	150	3.5	49	<0.001	<0.001
Logical Consistency	ChatTogoVar vs VarChat	150	2	208	<0.001	<0.001
Logical Consistency	GPT-4o vs VarChat	150	-1	2448	<0.001	0.001
Clarity and Conciseness	ChatTogoVar vs GPT-4o	150	3	0	<0.001	<0.001

Clarity and Conciseness	ChatTogoVar vs VarChat	150	1	179	<0.001	<0.001
Clarity and Conciseness	GPT-4o vs VarChat	150	-1	1538	<0.001	<0.001
Evidence Support	ChatTogoVar vs GPT-4o	150	7	0	<0.001	<0.001
Evidence Support	ChatTogoVar vs VarChat	150	1	438	<0.001	<0.001
Evidence Support	GPT-4o vs VarChat	150	-4	214	<0.001	<0.001

2-3. Manual (Secondary rater)

Evaluation Criterion	Comparison	Questions	Median Diff	WilcoxonTest		
				W	p-value	p-value (Holm)
Total	ChatTogoVar vs GPT-4o	150	19	94.5	<0.001	<0.001
Total	ChatTogoVar vs VarChat	150	11	406	<0.001	<0.001
Total	GPT-4o vs VarChat	150	-10	2701.5	<0.001	<0.001
Accuracy	ChatTogoVar vs GPT-4o	150	6	65.5	<0.001	<0.001
Accuracy	ChatTogoVar vs VarChat	150	5	311	<0.001	<0.001
Accuracy	GPT-4o vs VarChat	150	-1	4427.5	0.086	0.257
Completeness	ChatTogoVar vs GPT-4o	150	4	217.5	<0.001	<0.001
Completeness	ChatTogoVar vs VarChat	150	4	543.5	<0.001	<0.001
Completeness	GPT-4o vs VarChat	150	-1	4535	0.132	0.264
Logical Consistency	ChatTogoVar vs GPT-4o	150	1	224	<0.001	<0.001
Logical Consistency	ChatTogoVar vs VarChat	150	0	186	0.311	0.311
Logical Consistency	GPT-4o vs VarChat	150	-1	342	<0.001	<0.001
Clarity and Conciseness	ChatTogoVar vs GPT-4o	150	1	889	<0.001	<0.001

Clarity and Conciseness	ChatTogoVar vs VarChat	150	3	134	<0.001	<0.001
Clarity and Conciseness	GPT-4o vs VarChat	150	2	286	<0.001	<0.001
Evidence Support	ChatTogoVar vs GPT-4o	150	6	72	<0.001	<0.001
Evidence Support	ChatTogoVar vs VarChat	150	0	254.5	<0.001	<0.001
Evidence Support	GPT-4o vs VarChat	150	-7	25.5	<0.001	<0.001

2-4. LLM

Evaluation Criterion	Comparison	Questions	Median Diff	WilcoxonTest		
				W	p-value	p-value (Holm)
Total	ChatTogoVar vs GPT-4o	1500	15	44428	<0.001	<0.001
Total	ChatTogoVar vs VarChat	1500	15	6275	<0.001	<0.001
Total	GPT-4o vs VarChat	1500	5	456928	<0.001	<0.001
Accuracy	ChatTogoVar vs GPT-4o	1500	4	32389.5	<0.001	<0.001
Accuracy	ChatTogoVar vs VarChat	1500	3	5506	<0.001	<0.001
Accuracy	GPT-4o vs VarChat	1500	-1	487012	0.011	0.011
Completeness	ChatTogoVar vs GPT-4o	1500	3	58438.5	<0.001	<0.001
Completeness	ChatTogoVar vs VarChat	1500	3	6639.5	<0.001	<0.001
Completeness	GPT-4o vs VarChat	1500	1	436708	<0.001	<0.001
Logical Consistency	ChatTogoVar vs GPT-4o	1500	3	27612	<0.001	<0.001
Logical Consistency	ChatTogoVar vs VarChat	1500	2	2920	<0.001	<0.001
Logical Consistency	GPT-4o vs VarChat	1500	1	446634	<0.001	<0.001
Clarity and Conciseness	ChatTogoVar vs GPT-4o	1500	2	30892	<0.001	<0.001

Clarity and Conciseness	ChatTogoVar vs VarChat	1500	3	1074.5	<0.001	<0.001
Clarity and Conciseness	GPT-4o vs VarChat	1500	1	137101	<0.001	<0.001
Evidence Support	ChatTogoVar vs GPT-4o	1500	2	80075	<0.001	<0.001
Evidence Support	ChatTogoVar vs VarChat	1500	2	18280.5	<0.001	<0.001
Evidence Support	GPT-4o vs VarChat	1500	0	298848	<0.001	<0.001

Supplementary Table 3. Supplement to Table 5: Pairwise Wilcoxon Signed-Rank Tests Between Systems by Question Category

Post-hoc pairwise comparisons for Table 5 were performed on Total scores within each evaluation setting × question category using two-sided paired Wilcoxon signed-rank tests with Holm-adjusted p-values. Median paired differences were computed as (A − B), where A is the first system, and B is the second system listed in the "Comparison" column; positive values indicate higher scores for A than for B.

3-1. Manual (Mean of the primary and secondary raters)

				WilcoxonTest		
Question Category	Comparison	Questions	Median Diff	W	p-value	p-value (Holm)
Basic information	ChatTogoVar vs GPT-4o	23	23.5	0	<0.001	<0.001
Basic information	ChatTogoVar vs VarChat	23	9	0	<0.001	<0.001
Basic information	GPT-4o vs VarChat	23	-16.5	11	<0.001	<0.001
Allele frequency and population distribution	ChatTogoVar vs GPT-4o	21	17.5	1	<0.001	<0.001
Allele frequency and population distribution	ChatTogoVar vs VarChat	21	17.5	0	<0.001	<0.001
Allele frequency and population distribution	GPT-4o vs VarChat	21	1.5	81	0.230227	0.230227
Clinical significance	ChatTogoVar vs GPT-4o	23	23.5	1	<0.001	<0.001
Clinical significance	ChatTogoVar vs VarChat	23	6.5	40.5	0.00521975	0.00521975
Clinical significance	GPT-4o vs VarChat	23	-19.5	11.5	<0.001	<0.001
Pharmacogenomics	ChatTogoVar vs GPT-4o	16	17	0	<0.001	0.00131043
Pharmacogenomics	ChatTogoVar vs VarChat	16	9.25	2	<0.001	0.00131043

Pharmacogenomics	GPT-4o vs VarChat	16	-9.75	17	0.0145369	0.0145369
Functional impact	ChatTogoVar vs GPT-4o	25	20.5	0	<0.001	<0.001
Functional impact	ChatTogoVar vs VarChat	25	8.5	12.5	<0.001	<0.001
Functional impact	GPT-4o vs VarChat	25	-14	23	<0.001	<0.001
Evolutionary context	ChatTogoVar vs GPT-4o	13	22.5	0	<0.001	<0.001
Evolutionary context	ChatTogoVar vs VarChat	13	8.5	0	<0.001	<0.001
Evolutionary context	GPT-4o vs VarChat	13	-12.5	0	<0.001	<0.001
Comparison with related variants	ChatTogoVar vs GPT-4o	18	14.2	1	<0.001	<0.001
Comparison with related variants	ChatTogoVar vs VarChat	18	6.5	3.5	<0.001	<0.001
Comparison with related variants	GPT-4o vs VarChat	18	-7	12	0.00134109	0.00134109
Databases and Bioinformatics analysis	ChatTogoVar vs GPT-4o	11	12.5	0	<0.001	0.00292969
Databases and Bioinformatics analysis	ChatTogoVar vs VarChat	11	18	0	<0.001	0.00292969
Databases and Bioinformatics analysis	GPT-4o vs VarChat	11	2.5	19	0.425781	0.425781

3-2. Manual (Primary rater)

				WilcoxonTest		
Question Category	Comparison	Questions	Median Diff	W	p-value	p-value (Holm)
Basic information	ChatTogoVar vs GPT-4o	23	27	0	<0.001	<0.001
Basic information	ChatTogoVar vs VarChat	23	8	2	<0.001	<0.001
Basic information	GPT-4o vs VarChat	23	-18	18.5	<0.001	<0.001
Allele frequency and population distribution	ChatTogoVar vs GPT-4o	21	26	1.5	<0.001	<0.001
Allele frequency and population distribution	ChatTogoVar vs VarChat	21	21	0	<0.001	<0.001

Allele frequency and population distribution	GPT-4o vs VarChat	21	0	98.5	0.808185	0.808185
Clinical significance	ChatTogoVar vs GPT-4o	23	26	1	<0.001	<0.001
Clinical significance	ChatTogoVar vs VarChat	23	8	37.5	0.00381501	0.00381501
Clinical significance	GPT-4o vs VarChat	23	-17	4	<0.001	<0.001
Pharmacogenomics	ChatTogoVar vs GPT-4o	16	17.5	0	<0.001	0.00130174
Pharmacogenomics	ChatTogoVar vs VarChat	16	6.5	15	0.00604821	0.00623746
Pharmacogenomics	GPT-4o vs VarChat	16	-11	8	0.00311873	0.00623746
Functional impact	ChatTogoVar vs GPT-4o	25	26	0	<0.001	<0.001
Functional impact	ChatTogoVar vs VarChat	25	5	18	<0.001	<0.001
Functional impact	GPT-4o vs VarChat	25	-17	1	<0.001	<0.001
Evolutionary context	ChatTogoVar vs GPT-4o	13	24	0	<0.001	<0.001
Evolutionary context	ChatTogoVar vs VarChat	13	7	0	<0.001	<0.001
Evolutionary context	GPT-4o vs VarChat	13	-15	1	<0.001	<0.001
Comparison with related variants	ChatTogoVar vs GPT-4o	18	10	0	<0.001	<0.001
Comparison with related variants	ChatTogoVar vs VarChat	18	5	0	<0.001	<0.001
Comparison with related variants	GPT-4o vs VarChat	18	-5	7	<0.001	<0.001
Databases and Bioinformatics analysis	ChatTogoVar vs GPT-4o	11	16	0	<0.001	0.00292969
Databases and Bioinformatics analysis	ChatTogoVar vs VarChat	11	25	0	<0.001	0.00292969
Databases and Bioinformatics analysis	GPT-4o vs VarChat	11	8	9.5	0.0351562	0.0351562

3-3. Manual (Secondary rater)

Question Category	Comparison	Questions	Median Diff	WilcoxonTest		
				W	p-value	p-value (Holm)
Basic information	ChatTogoVar vs GPT-4o	23	20	0	<0.001	<0.001
Basic information	ChatTogoVar vs VarChat	23	10	0	<0.001	<0.001
Basic information	GPT-4o vs VarChat	23	-11	24.5	<0.001	<0.001
Allele frequency and population distribution	ChatTogoVar vs GPT-4o	21	9	2	<0.001	<0.001
Allele frequency and population distribution	ChatTogoVar vs VarChat	21	13	0	<0.001	<0.001
Allele frequency and population distribution	GPT-4o vs VarChat	21	4	63	0.0653354	0.0653354
Clinical significance	ChatTogoVar vs GPT-4o	23	23	0	<0.001	<0.001
Clinical significance	ChatTogoVar vs VarChat	23	3	51.5	0.0147697	0.0147697
Clinical significance	GPT-4o vs VarChat	23	-20	32	0.00125845	0.0025169
Pharmacogenomics	ChatTogoVar vs GPT-4o	16	19	1	0.00120803	0.00241606
Pharmacogenomics	ChatTogoVar vs VarChat	16	12.5	1	<0.001	0.0023458
Pharmacogenomics	GPT-4o vs VarChat	16	-10	26	0.0494572	0.0494572
Functional impact	ChatTogoVar vs GPT-4o	25	19	1	<0.001	<0.001
Functional impact	ChatTogoVar vs VarChat	25	13	25.5	<0.001	0.00109725
Functional impact	GPT-4o vs VarChat	25	-10	73	0.0158133	0.0158133
Evolutionary context	ChatTogoVar vs GPT-4o	13	19	1	<0.001	<0.001
Evolutionary context	ChatTogoVar vs VarChat	13	11	0	<0.001	<0.001

Evolutionary context	GPT-4o vs VarChat	13	-10	14	0.0244141	0.0244141
Comparison with related variants	ChatTogoVar vs GPT-4o	18	20.5	8.5	<0.001	0.00236754
Comparison with related variants	ChatTogoVar vs VarChat	18	10.5	9.5	0.00243707	0.00487414
Comparison with related variants	GPT-4o vs VarChat	18	-10	35	0.0255738	0.0255738
Databases and Bioinformatics analysis	ChatTogoVar vs GPT-4o	11	15	1.5	0.00292969	0.00585938
Databases and Bioinformatics analysis	ChatTogoVar vs VarChat	11	9	0	<0.001	0.00292969
Databases and Bioinformatics analysis	GPT-4o vs VarChat	11	-6	23	0.400391	0.400391

3-4. LLM

				WilcoxonTest		
Question Category	Comparison	Questions	Median Diff	W	p-value	p-value (Holm)
Basic information	ChatTogoVar vs GPT-4o	240	15	868.5	<0.001	<0.001
Basic information	ChatTogoVar vs VarChat	240	20	0	<0.001	<0.001
Basic information	GPT-4o vs VarChat	240	10	8997.5	<0.001	<0.001
Allele frequency and population distribution	ChatTogoVar vs GPT-4o	210	10	4193.5	<0.001	<0.001
Allele frequency and population distribution	ChatTogoVar vs VarChat	210	20	0	<0.001	<0.001
Allele frequency and population distribution	GPT-4o vs VarChat	210	15	372	<0.001	<0.001
Clinical significance	ChatTogoVar vs GPT-4o	240	15	776.5	<0.001	<0.001
Clinical significance	ChatTogoVar vs VarChat	240	8	1311.5	<0.001	<0.001
Clinical significance	GPT-4o vs VarChat	240	-5	9593	<0.001	<0.001
Pharmacogenomics	ChatTogoVar vs GPT-4o	150	15	817.5	<0.001	<0.001
Pharmacogenomics	ChatTogoVar vs VarChat	150	15	91	<0.001	<0.001

Pharmacogenomics	GPT-4o vs VarChat	150	5	3764.5	<0.001	<0.001
Functional impact	ChatTogoVar vs GPT-4o	210	15	121.5	<0.001	<0.001
Functional impact	ChatTogoVar vs VarChat	210	8	0	<0.001	<0.001
Functional impact	GPT-4o vs VarChat	210	-5	4071.5	<0.001	<0.001
Evolutionary context	ChatTogoVar vs GPT-4o	150	15	101	<0.001	<0.001
Evolutionary context	ChatTogoVar vs VarChat	150	10	0	<0.001	<0.001
Evolutionary context	GPT-4o vs VarChat	150	-5	4800	0.0973316	0.0973316
Comparison with related variants	ChatTogoVar vs GPT-4o	180	15	0	<0.001	<0.001
Comparison with related variants	ChatTogoVar vs VarChat	180	10	71	<0.001	<0.001
Comparison with related variants	GPT-4o vs VarChat	180	-5	5337	<0.001	<0.001
Databases and Bioinformatics analysis	ChatTogoVar vs GPT-4o	120	10.5	469.5	<0.001	<0.001
Databases and Bioinformatics analysis	ChatTogoVar vs VarChat	120	20	0	<0.001	<0.001
Databases and Bioinformatics analysis	GPT-4o vs VarChat	120	10	338.5	<0.001	<0.001

Supplementary Table 4. Inter-Rater Agreement for Manual Total Scores (Between primary and secondary raters)

This table summarizes inter-rater agreement for the manual Total score assigned by two independent raters. Agreement is reported pooled across all systems (Pooled; n = 150 questions × 3 systems = 450) and stratified by system (n = 150 per system). We report ICC(2,1) (two-way random-effects, absolute-agreement, single-rater reliability) and ICC(2,2) (reliability of the mean of two raters' scores), along with the quadratic-weighted Cohen's κ. Absolute differences between raters' total scores are summarized as mean, median, and maximum of |Δ| (abs(delta)).

System	Questions	ICC(2,1)	ICC(2,2)	Weighted κ (quadratic)	abs(delta) mean	abs(delta) median	abs(delta) max
Pooled (all systems)	450	0.686	0.814	0.686	6.629	5	28
ChatTogoVar	150	0.22	0.361	0.22	6.193	5.5	19
GPT-4o	150	0.396	0.568	0.391	7.687	7	25
VarChat	150	0.493	0.66	0.494	6.007	5	28

Supplementary Table 5. Manual and LLM-Based Evaluation Scores for Answers to a Sample Question

Sample Question: What is the clinical significance of rs34637584 in ClinVar?

Evaluation		Mean score					
Setting	System	Total	Accuracy	Completeness	Logical Consistency	Clarity and Conciseness	Evidence Support
Manual (mean)	ChatTogoVar	47	9.5	9.5	9.5	9.5	9
Manual (mean)	GPT-4o	17.5	1	2.5	6.5	6.5	1
Manual (mean)	VarChat	37	7.5	7.5	8	6	8
LLM	ChatTogoVar	45	10	9	10	9	7
LLM	GPT-4o	30	5	6	7	7	5
LLM	VarChat	20	4	5	5	3	3
